# The relationship of tongue fat content and efficacy of uvulopalatopharyngoplasty in Chinese patients with obstructive sleep apnea

**DOI:** 10.1186/s12893-023-02144-x

**Published:** 2023-08-27

**Authors:** Bingjie Zhao, Zine Cao, Yushan Xie, Yewen Shi, Yitong Zhang, Shiyu Liu, Xi Chen, Lina Ma, Xiaoxin Niu, Yonglong Su, Yani Feng, Chunfeng Lian, Xiaoyong Ren, Haiqin Liu

**Affiliations:** 1 Department of Otorhinolaryngology Head and Neck Surgery, The Second Affiliated Hospital of Xi’an Jiaotong University, No.157, Xiwu Road, Xi’an, Shaanxi 710004 China; 2 School of Mathematics and Statistics, Xi’an Jiaotong University, No.28, Xianningxi Road, Xi’an, Shaanxi 710049 China

**Keywords:** Obstructive sleep apnea, Tongue fat, Magnetic resonance imaging, Uvulopalatopharyngoplasty, Upper airway

## Abstract

**Background:**

To investigate the relationship between tongue fat content and severity of obstructive sleep apnea (OSA) and its effects on the efficacy of uvulopalatopharyngoplasty (UPPP) in the Chinese group.

**Method:**

Fifty-two participants concluded to this study were diagnosed as OSA by performing polysomnography (PSG) then they were divided into moderate group and severe group according to apnea hypopnea index (AHI). All of them were also collected a series of data including age, BMI, height, weight, neck circumference, abdominal circumference, magnetic resonance imaging (MRI) of upper airway and the score of Epworth Sleepiness Scale (ESS) on the morning after they completed PSG. The relationship between tongue fat content and severity of OSA as well as the association between tongue fat content in pre-operation and surgical efficacy were analyzed.Participants underwent UPPP and followed up at 3^rd^ month after surgery, and they were divided into two groups according to the surgical efficacy.

**Results:**

There were 7 patients in the moderate OSA group and 45 patients in the severe OSA group. The tongue volume was significantly larger in the severe OSA group than that in the moderate OSA group. There was no difference in tongue fat volume and tongue fat rate between the two groups. There was no association among tongue fat content, AHI, obstructive apnea hypopnea index, obstructive apnea index and Epworth sleepiness scale (all *P* > 0.05), but tongue fat content was related to the lowest oxygen saturation (r=-0.335, *P <* 0.05). There was no significantly difference in pre-operative tongue fat content in two different surgical efficacy groups.

**Conclusions:**

This study didn’t show an association between tongue fat content and the severity of OSA in the Chinese group, but it suggested a negative correlation between tongue fat content and the lowest oxygen saturation (LSaO_2_). Tongue fat content didn’t influence surgical efficacy of UPPP in Chinese OSA patients.

**Trial registration:**

This study didn’t report on a clinical trial, it was retrospectively registered.

**Supplementary Information:**

The online version contains supplementary material available at 10.1186/s12893-023-02144-x.

## Background

Obstructive Sleep Apnea (OSA) refers to a common sleep disorder that results in hypopnea and hypoxemia caused by repeated collapse of the upper airway during sleep [1]. In the latest epidemiological study, China has the largest number of affected individuals of OSA in the global, with a prevalence of about 23.6% in people aged 30–69 years old. [2] OSA is a main cause of excessive sleepiness which effects quality of life and high risk in vehicle crash. And it is associated with increase in incidence for various diseases such as hypertension, type 2 diabetes mellitus, atrial fibrillation and stroke. [1] Effective treatments for OSA include behavioral measures, medical devices (e.g. positive airway pressure and oral appliances) and surgery. [1] The most widely applied surgical procedure in OSA is uvulopalatopharyngoplasty (UPPP), but the surgical efficiency is 40-50% which implys that we need strict surgical indications. [3].

The development of the obesity epidemic is likely to contribute to the rising of the incidence of OSA. [2,4,5] The characteristic of fat deposition in OSA patients is imbalanced which means fat often distribute around the upper airway. [6–8] Tongue is the largest muscular organ, mainly composed of the largest dilator of the upper airway – genioglossus. [9–11] The fat deposition in the tongue can contribute to the disorder of enlargement of the upper airway, this may also affect efficacy of UPPP. Previous study had shown that tongue fat volume correlated with apnea-hypopnea index (AHI) and body mass index (BMI), and hypothesized the tongue fat volume could link obesity to OSA. [12] However, because of differences in races, there is a few numbers of researches in relationships between tongue fat content and the severity of OSA in Chinese group. And a research suggested that there were no significant post-volumetric changes of upper airway including tongue volume in 6 months post-surgery, [13] but it is also not clear if fat content influence surgery outcomes.

The purpose of this study was to explore:1) the correlation between tongue fat content and the severity of OSA; 2) the association between tongue fat content in pre-operation and surgical efficacy, whether tongue fat content could be considered as a predicator of surgical treatment.

## Methods

### Participants

This is a retrospective study. These patients who were from the department of otolaryngology of the Second Affiliated Hospital of Xi’an Jiao tong University, China from February 24^th^ to December 8^th^ in 2021. They were diagnosed as OSA defined by AHI ≥ 5 by performing polysomnography (PSG). And they were collected a series of data on the morning after they were performed PSG, which included age, BMI, height, weight, neck circumference, abdominal circumference, magnetic resonance imaging (MRI) of upper airway and the score of Epworth Sleepiness Scale (ESS). [1] They also did the Müller test under the laryngoscopy which results showed that their upper airway was predominantly collapsed in the palatal region. All participants were evaluated by 2–3 senior otolaryngology doctors to confirm the feasibility for surgery. The indications of surgery were according to the Chinese Multidisciplinary guidelines of OSA (2018) and the SAMS clinical trial. [14,15] We included participants based on the following criteria. The eligibility criteria was age 18–50 years, BMI < 32 kg/m^2^, Friedman stage I-II and the score of ESS > 8 [16]. The exclusion criteria was prior surgery on palate, tongue, mandible or maxilla, nasal obstruction uncontrolled by medication or surgery, clinically significant retrognathia (confirmed by lateral skull x-ray), and other chronic diseases including moderate to severe COPD (FEV/FVC ratio < 70% and FEV 1 < 50%), heart failure, cardiovascular and cerebrovascular diseases, chronic narcotic use, major depression (e.g. hospitalisation for depression, suicide attempt or symptoms necessitating antidepressant drug dose escalation in the previous 3 months), unacceptable anaesthetic or surgical risk (e.g. anticoagulant or antiplatelet medication which cannot be withdrawn). Written informed consents from participants were obtained.

There were very few patients with mild OSA who could meet the above criteria, and most of them chose non-surgical treatment such as weight loss. So there were no suitable patients with mild OSA could be included in this study. Since it was a retrospective research, we didn’t include non-OSA participants.

### PSG

Participants accepted in-laboratory PSG (Alice 6, PHILIPS, America) according to the guide of American Academy of Sleep Medicine at night, and the studying started from lights-off and ended in lights-on. [17] The parameters included AHI, obstructive apnea hypopnea index (OAHI), obstructive apnea index (OAI), the lowest oxygen saturation (LSaO_2_). All test results are interpreted by two sleep medicine technologists. Participants were divided into moderate OSA group and severe OSA group according to AHI (15–29 indicates moderate OSA and ≥ 30 indicates severe OSA).

### Surgery intervention

Standardized surgery called uvulopalatopharyngoplasty (UPPP) was performed on every participants. [18] The amount of tongue fat content was blind to operators. The procedure removed the palatine tonsils, part of fat pads, mucosa of lateral pharyngeal and uvula. Additionally, the anterior and posterior arches were fixed with apposition suture and the uvula was remodeled. In this procedure, no more measures on the tongue were performed.

Patients were performed PSG at a 3-month follow-up visit. In this study, the successful surgical treatment was defined as a > 50% reduction in AHI according to a meta-analysis which we thought it was more representative. [19] Patients who received follow-ups were divided into two groups namely responders (AHI reduced by > 50%) and non-responders (AHI reduced by ≤ 50%).

### MRI analysis

Upper airway imaging studies were performed by using 3T MRI scanner (GE medical system SIGNA Pioneer) according to a standardized scanning protocol. Participants were instructed to lie in supine position, breathe through noses and refrain from swallowing. Contiguous T1-weighted spin-echo axial and sagittal images were obtained to confirm the boundary of tongue. IDEAL sequence scanning was added to manifest the fat of tongue, which could overcome the influence of magnetic field in homogeneity and separate water and fat thoroughly than Dixon sequence.

Image analysis was performed by ITK-SNAP (Version 3.8.0). In this study, the segmentation of tongue fat content (tongue fat volume and tongue fat rate) was marked by people. It was finished by a single professional with the measurement reproducibility confirmed.

**Fig. 1:**
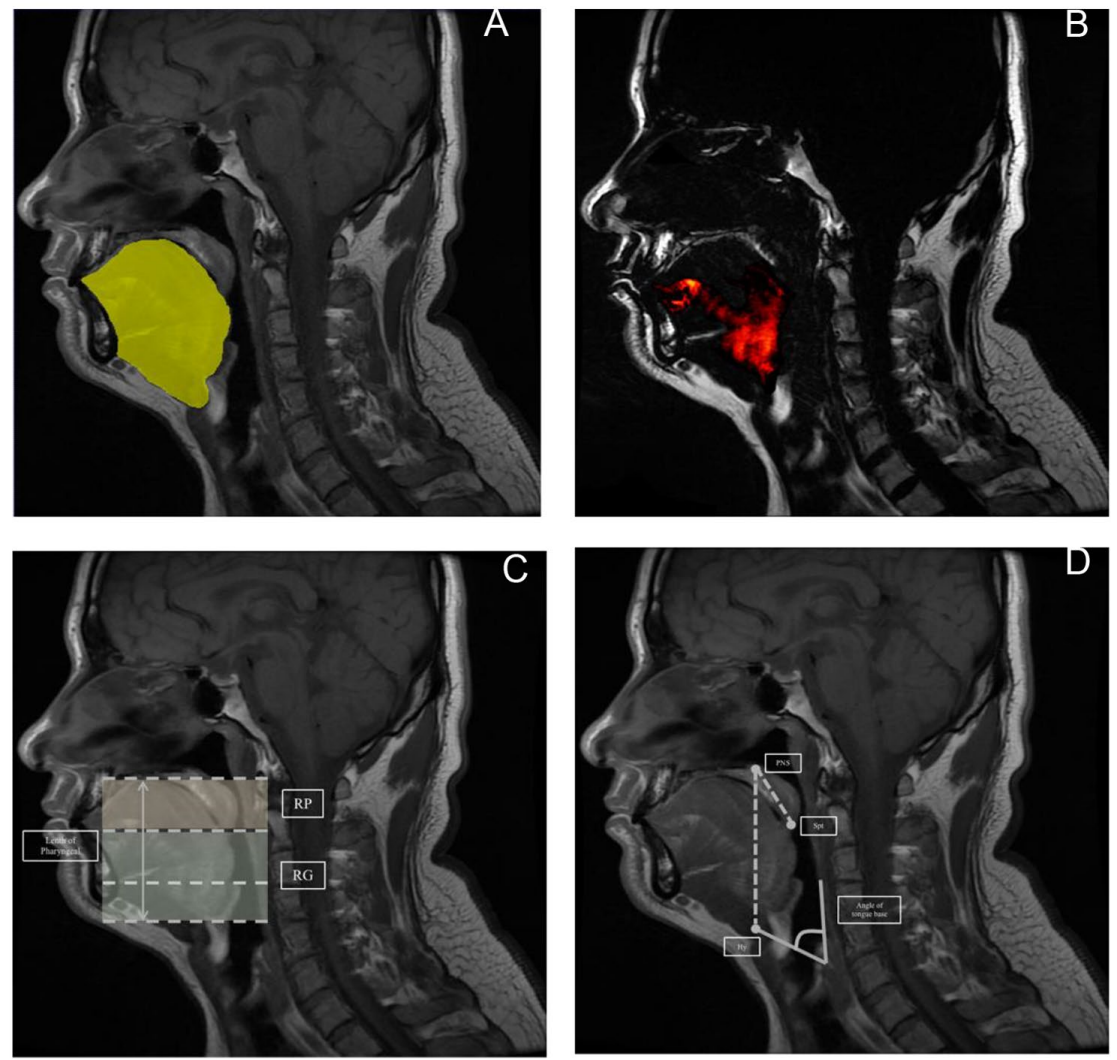
Magnetic resonance imaging analysis. **A)** Sagittal view of segmentation in tongue with segmentation of tongue (area in yellow). **B)** The region of tongue fat which is marked in red. **C)** Upper airway is divided into two parts: Retropalatal (RP) area is from the level of hard palate to the tip of uvula (transparent yellow); Retroglossal (RG) area is from the level of the tip of uvula to the base of epiglottis (transparent green). **D)** The diagram of landmarks which are used to calculate the length from posterior nasal spine (PNS) to hyoid (Hy) and soft palate tip (SPt). The angle of tongue base is defined as the inferior margin of geniohyoid and the posterior of pharyngeal wall

The basic anatomical figures including pharyngeal length (between the hard palate and epiglottis base) which was divided into two parts according to the uvula tip, airway cross-sectional area (CSA) (the narrowest area, nasopharynx and oropharynx), velopharynx length was from the hard palate and the end of the soft palate (SPt), oropharynx length was from the SPt to the base of the epiglottis, pharynx length was the sum of velopharynx one and oropharynx one, soft palate length was from the posterior nasal spine (PNS) to the SPt, length from PNS to the hyoid bone (Hy) and the angle of tongue base. Upper airway was divided into two areas: Retropalatal (RP) area was from the level of hard palate to the tip of uvula; Retroglossal (RG) area was from the level of the tip of uvula to the base of epiglottis. All of these figures were performed on mid-sagittal images (Fig. 1).

### Statistical analysis

Statistical analysis was performed by using SPSS (version 26.0, IBM Corporation). Intraclass correlation coefficients were computed to assess the reproducibility of tongue fat content. Continuous variables were assessed for normality of distribution. The baseline demographics of responders and non-responders’ groups were compared by using independent t-tests or Mann-Whitney tests.

## Results

### Participant characteristics

Totally 52 participants were concluded according to inclusion/exclusion criteria. 24 of the 52 participants were followed up by PSG, and 14 were responders. Their upper airway mainly collapsed in the palatal region according to the laryngological examination combined with Müller test. There were no significant differences in all variables between responders and non-responders. On average, participants were overweight (BMI 26.78 ± 2.84 kg/m^2^) with severe OSA (AHI 57.73 ± 21.65 events/hour) (Table 1).

**Table 1:** Characteristics of all participants

	All participants	The severity of OSA		*P*	Follow-up participants		*P*
		Moderate	Severe		Responder	Non-responder	
N	52	7	45		14	10	-
Age (years)	35.55 ± 7.32	32.29 ± 5.94	36.07 ± 7.44	> 0.05	37.10 ± 5.43	36.14 ± 7.95	> 0.05
BMI (kg/m^2^)	26.78 ± 2.84	24.86 ± 2.23	27.08 ± 2.83	> 0.05	25.57 ± 2.38	26.96 ± 2.60	> 0.05
Height (cm)	172.65 ± 4.60	175.00 ± 7.00	172.27 ± 4.09	> 0.05	173.20 ± 3.97	173.64 ± 4.62	> 0.05
Weight (kg)	79.75 ± 8.30	75.79 ± 3.05	80.38 ± 8.71	> 0.05	76.40 ± 6.90	81.21 ± 8.16	> 0.05
Neck circumference (cm)	40.08 ± 2.12	38.57 ± 2.30	40.32 ± 2.02	> 0.05	39.40 ± 2.27	40.00 ± 2.15	> 0.05
Abdominal circumference (cm)	97.82 ± 7.91	92.86 ± 4.60	98.61 ± 8.07	> 0.05	96.20 ± 7.05	97.14 ± 6.55	> 0.05
AHI (events/hour)	57.73 ± 21.65	21.54 ± 4.52	63.49 ± 17.17	< 0.05^*^	47.92 ± 14.93	55.47 ± 21.70	> 0.05
OAHI (events/hour)	52.18 ± 20.04	20.58 ± 5.01	57.20 ± 16.59	< 0.05^*^	47.36 ± 14.79	52.30 ± 20.21	> 0.05
OAI (events/hour)	40.09 ± 23.76	6.89 ± 7.28	44.38 ± 21.00	< 0.05^*^	30.16 ± 19.49	45.05 ± 27.02	> 0.05
LSaO_2_ (%)	69.88 ± 10.71	85.14 ± 4.63	67.45 ± 9.30	< 0.05^*^	72.80 ± 8.64	70.00 ± 9.17	> 0.05
ESS	10.82 ± 4.88	7.86 ± 4.88	11.30 ± 4.77	> 0.05	10.50 ± 5.46	10.71 ± 5.51	> 0.05

*indicates statistical significance

*BMI = body mass index; AHI = apnea hypopnea index; OAHI = obstructive apnea hypopnea index; OAI = obstructive apnea index; LSaO*_*2*_ *= lowest oxygen saturation; ESS = Epworth sleepiness*

### Measurement reproducibility

The segmentation of intra-tongue fat was performed by the same operator at intervals of six months. Intra-rater reliability was assessed by using the intraclass correlation coefficient (ICC). In this study, the ICC was 0.84 which means the reliability and reproducibility of manual data are excellent (n = 52).

### Characteristics of tongue fat content in group of OSA

**Fig. 2:**
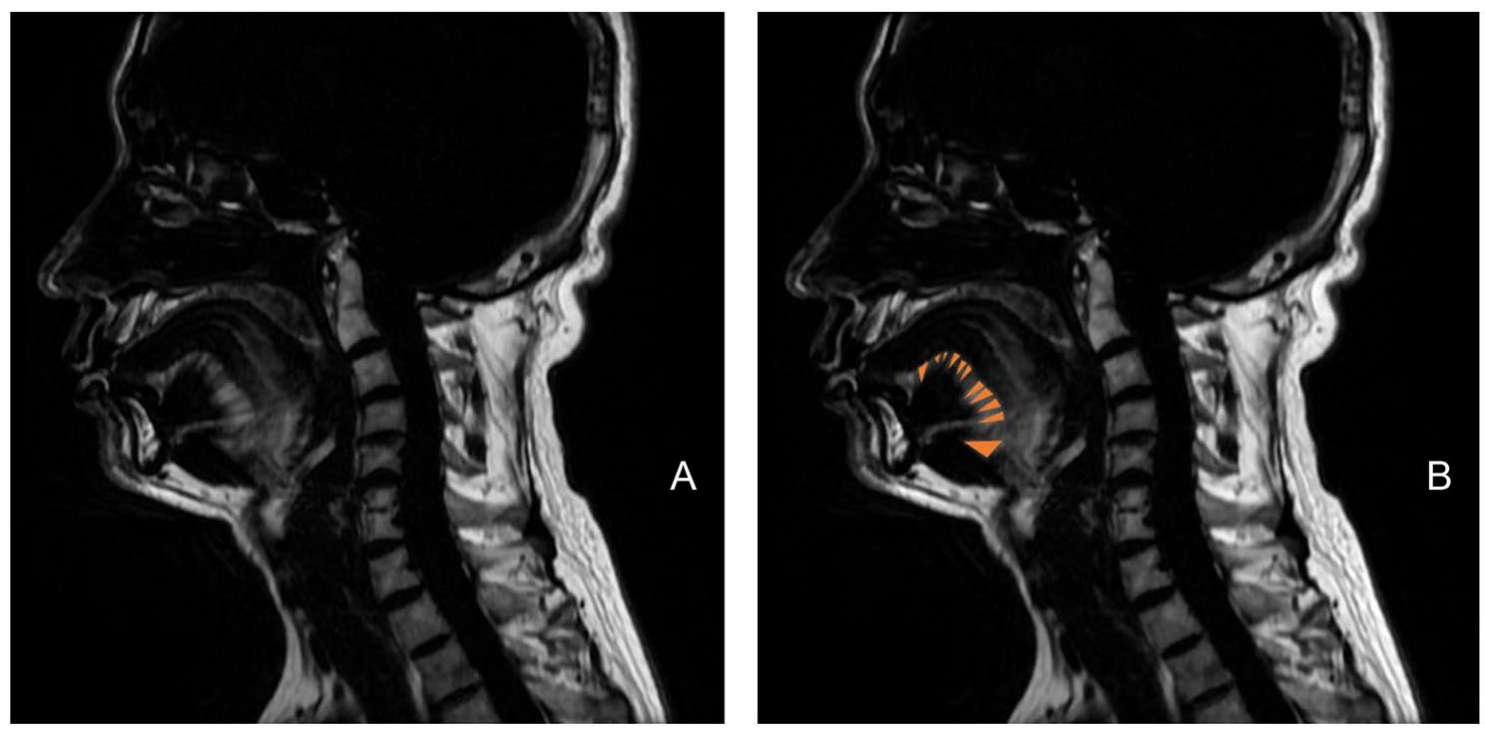
Characteristics of tongue fat. The sagittal slice of MRI in tongue fat which characteristics of distribution are fanned out **(A)**, fat was marked by yellow **(B)**

The whole tongue volume of all participants was (116.01 ± 19.04) cm^3^, and that in moderate and severe patients were (97.29±29.34) cm^3^ and (118.98±15.34)cm^3^ respectively. The total fat volume and fat rate of 52 patients were (10.37 ± 2.74) cm^3^ and (9.03 ± 2.14)% respectively (Table 2). Fat content was higher in RG region than that in RP region. The image of tongue fat in the mid-sagittal slice of IDEAL image was fanned out (Fig. 2).

**Table 2:** Charateristics of pre-operative fat content in moderate and severe OSA patients

	Total	The severity of OSA		*P*
		Moderate	Severe	
Volume of tongue (cm^3^)	116.01 ± 19.04	97.29±29.34	118.98±15.34	< 0.05^*†^
Fat volume of tongue (cm^3^)				
Total	10.37 ± 2.74	8.46±2.78	10.68±2.64	> 0.05
RP region	2.64 ± 2.83	1.59±2.09	2.81±2.91	> 0.05^*†*^
RG region	7.73 ± 2.99	6.87±2.76	7.87±3.03	> 0.05
Fat rate (%)				
Total	9.03 ± 2.14	9.07±2.60	9.02±2.10	> 0.05
RP region	2.29 ± 2.33	1.95±2.32	2.34±2.36	> 0.05^*†*^
RG region	6.74 ± 2.37	7.13±1.78	6.68±2.46	> 0.05

RP: Retropalatal; RG: Retroglossal

^*^:indicates statistical significance, ^*†*^: These variables were compared by Mann-Whitney tests. Other variables were compared by t-tests.

Higher whole tongue volume was associated with higher tongue fat volume (r = 0.318, *P <* 0.05). Higher tongue fat volume was associated with tongue fat rate (r = 0.308, *P <* 0.05) in RG region. We also analysed relationships between tongue fat content and clinical factors in this group. Higher whole tongue volume was associated with higher BMI (r = 0.377, *P* < 0.05), higher weight (r = 0.478, *P* < 0.05), longer neck circumference (r = 0.445, *P* < 0.05), longer abdominal circumference (r = 0.487, *P* < 0.05) (see Figure S1 in the supplemental material).

### Relationship between the severity of OSA and intra-tongue fat in Chinese

**Fig. 3:**
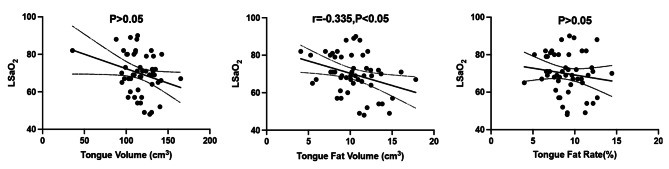
Correlations between indicators of intra-tongue fat and LSaO_2_. 95% confidence intervals are plotted on each graph. LSaO_2_: lowest oxygen saturation

There was significant difference in the tongue volume between moderate and severe patients (*P* < 0.05) (Table 2), but there was no statistical difference in the tongue fat content between these two groups. Tongue fat volume was negative correlated to the level of LSaO_2_ (r = − 0.335, *P* < 0.05) (Fig. 3). There were no linear correlations among tongue fat content, the severity of OSA and ESS (see Figure S2 in the supplemental material).

### The relationship between pre-operative tongue fat content and surgery efficacy

By comparing pre-operative tongue fat content between the responders and non-responders, there was no significant difference between these two groups. The same result was shown in the percentage of intra-tongue fat. The details were shown in the Table 3.

**Table 3:** Tongue fat volume and fat rate of responders versus non-responders

	Follow-up participants		*P*
	Responder	Non-responder	
Fat volume of tongue (cm^3^)			
Total	11.22 ± 2.81	9.88 ± 2.38	0.205
RP region	2.20 ± 2.34	2.63 ± 3.07	0.979^†^
RG region	9.01 ± 2.76	7.25 ± 2.89	0.136
Fat rate (%)			
Total	9.29 ± 1.28	8.73 ± 2.51	0.460
RP region	1.81 ± 1.88	2.19 ± 2.38	0.769^†^
RG region	7.48 ± 1.91	6.54 ± 2.78	0.317

RP: Retropalatal; RG: Retroglossal

^*†*^: These variables were compared by Mann-Whitney tests. Other variables were compared by t-tests

### Correlations

**Fig. 4:**
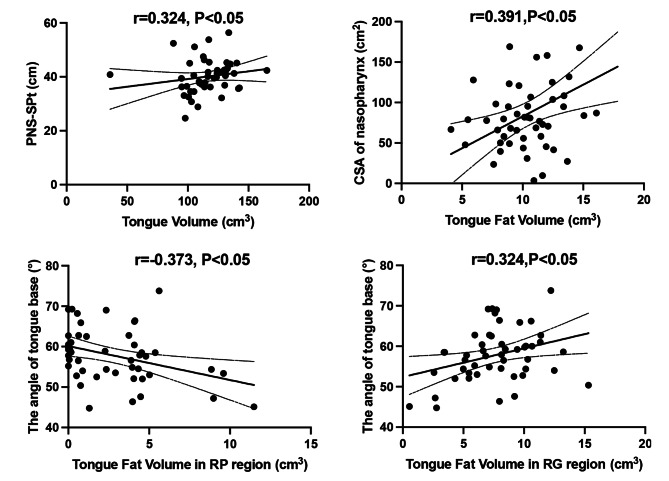
Correlations between related indicators of tongue fat and other anatomical measurements. 95% confidence intervals are plotted on each graph. PNS-SPt: length from posterior nasal spine to soft palate tip; CSA: Cross-sectional area; RP: Retropalatal; RG: Retroglossal

There was no significantly difference between moderate group and severe group in anatomical measurements (Table 4). Then we analyzed correlations between indicators of tongue fat content and other anatomical measurements (Fig. 4).

**Table 4:** Anatomical measurements of moderate and severe group in OSA

	Total	The severity of OSA		*P*
		Moderate	Severe	
Cross-sectional area (cm^3^)				
The narrowest area	32.47±20.16	29.65±8.14	32.94±21.55	> 0.05
Nasopharynx	86.19±55.76	84.11±38.56	86.54±58.49	> 0.05
Oropharynx	160.41±72.26	159.53±60.97	160.56±74.63	> 0.05
Length (cm)				
PNS-Hy	81.53±7.33	79.21±5.58	81.92±7.57	> 0.05
PNS-SPt	40.12±6.14	42.56±6.27	39.71±6.10	> 0.05
Pharynx	57.53±7.61	59.42±6.70	57.22±7.78	> 0.05
Velopharynx	34.73±5.61	36.57±7.63	42.04±7.34	> 0.05
Oropharynx	45.50±9.47	42.04±7.34	46.08±9.74	> 0.05
The angle of tongue base (°)	57.86±6.59	56.30±6.91	58.12±6.58	> 0.05

PNS-Hy: the length from posterior nasal spine (PNS) to hyoid (Hy); PNS-SPt: the length from posterior nasal spine (PNS) to soft palate tip (SPt)

Higher tongue volume was positively associated with longer length of PNS-SPt (r = 0.324, *P* < 0.05). Higher tongue fat volume was positively associated with larger cross-sectional area of nasopharynx (r = 0.391, *P* < 0.05). Higher tongue fat volume in RP region was negatively related to larger angle of tongue base, and higher tongue fat volume in RG region was positively related to larger angle of tongue base (r=-0.373, *P* < 0.05; r = 0.324, *P* < 0.05).

## Discussion

The former study showed that the type of disproportion between tongue volume and craniofacial framework will cause increased tissue pressure around the upper airway, and lead to the closure of the pharyngeal airway. [20] Kim et. al’s study showed that tongue fat played an important role in increasing tongue volume. [12] Studies about the measurement on the tongue-fat in Chinese and related data was less. [21–23] So the objective of this study was to determine whether the intra-tongue fat was related to the severity of OSA and had effects on surgery in Chinese group (participants came from Chinese mainland).

The study showed that tongue fat volume and tongue fat rate were 32.97 ± 9.18 cm^3^ and 32.6 ± 7.9% in Caucasian and African American people. [12] In our study, Chinese people had lower tongue fat volume (10.37 ± 2.74 cm^3^) and fat rate (9.03 ± 2.14%). This may be due to lower BMI range (26.78 ± 2.84 kg/m^2^) of the participants recruited than that study (34.1 ± 4.8 kg/m^2^).^12^ In another research, the average BMI was close to 27 kg/m^2^ and the tongue fat percentage was 17.9 ± 4.6% which was still higher than our study. [24] It’s necessary to do related research in Chinese group because of inter-ethnic differences.

There were no significant correlations among the tongue fat content and AHI, OAHI and OAI. This result was different from that in Kim et al.’s study (Both tongue volume and tongue fat volume had significant positive correlations with AHI). [12] However, the higher tongue fat volume was related to the lower LSaO_2_. This may closely related to hypoxia inducible factor-1α (HIF-1α) which is a vital hypoxia-induced chemokine, and it can mediate overall adipose tissue inflammation by participating in process of insulin resistance and glucose intolerance. [25,26] It may explain why tongue fat volume was associated with hypoxia index. And maybe we should focus on more hypoxia indicators including LSaO_2_ when we assess the severity of OSA rather than only using AHI. [27].

Another finding of our study was that whole tongue volume was positively related to BMI, but tongue fat rate was not related to BMI, which was not consistent to the result of Jugé et al.’s study. [24] The tongue volume was larger in severe group than in moderate group, and it was the same as Schwab et al.’s study and Jugé et al.’s study. [24,28] Tongue volume was positively related to neck circumference and abdominal circumference, maybe tongue volume could contribute to more severe OSA besides the latter two factors. [29] These results may suggest that tongue size is more important than fat content in patients with OSA in Chinese group, and it is consistent with previous hypotheses. [6,7] Perhaps the large tongue volume in the Chinese population is not due to an increase in tongue fat volume, but is due to edema and inflammation of the muscle tissue mostly.

There are several methods recommended to predict surgical efficacy including Friedman staging system and TCM scoring system. [30,31] Although performing UPPP on patients who are Friedman stage I, the surgical success rates could not reach at a good level. [32] To enhance the success rate of surgery, the further study of surgical indications for patients with OSA is important. In this study, there was no difference was seen in tongue fat content of baseline between patients with responders and non-responders to UPPP. It may suggest that although non-visible tongue fat content may contribute to tongue retropulsion, it was not an important factor to surgical efficacy in this cohort of patients who were eligible for surgery. That was consistent to the main result of Sutherland et al.’s study which showed there was no association between anatomical changes (tongue volume and tongue fat volume) and AHI improvement. [13] This result was the same after divided by anatomical regions (RP and RG region). Tongue is a motor organ which is comprised by muscle entirely, some research suggested that tongue muscle activity is higher during wakefulness and would diminish to a greater extent during sleep in patients with OSA, especially in REM sleep. [33] Fatigue of muscle and change of shape at night may contribute to a greater impact in tongue retropulsion than tongue fat content. [34] In the future, we could try to simulate the state of the upper airway during sleep by using Muller test and acquire MRI images. This may help us to understand tongue shape and tongue fat distribution at night then we can explore the impact of tongue fat to tongue retropulsion. In addition, maybe we need other methods like computer methods to manifest the characteristics of fat deposition in tongue without the limit of traditional anatomy which defined by retropalatal and retroglossal region.

Our limitations involve: (1) Our study population was small, we could not generalize the same conclusions to women since our participants were all male. Because of low prevalence in female, OSA has been considered as a male disease with male: female ratios ranging from 3:1 to 5:1 in the general population. [35] Future studies should collect more patients to enrich the population of different genders, ages and builds. (2) We didn’t recruit people with no-OSA as controls to analyse. (3) Because of the limitation in surgical indications, participants were mainly moderate and severe patients with OSA. (4) Since there was no uniform standard for professional to mark tongue fat region, there may be some errors in the results. So it is important to improve the accuracy of segmentations in tongue fat, we would accumulate abundant numbers of images to apply computer algorithm such as deep learning in segmentation. It can obtain much more accurate figure about this structure.

## Conclusions

This study didn’t show an association between tongue fat content and the severity of OSA in the Chinese group, but it suggested a negative correlation between tongue fat content and the lowest oxygen saturation (LSaO_2_). Tongue fat content didn’t influence surgical efficacy of UPPP in Chinese OSA patients.

### Electronic supplementary material

Below is the link to the electronic supplementary material.

Additional File 1: Figure s1 and s2

## Data Availability

The datasets generated and/or analysed during the current study are not publicly available due individual privacy could be compromised but are available from the corresponding author on reasonable request.

## List of Abbreviations

OSA: Obstructive sleep apnea
UPPP: Uvulopalatopharyngoplasty
PSG: Polysomnography
MRI: Magnetic resonance imaging
AHI: Apnea-hypopnea index
LSaO2: The lowest oxygen saturation
BMI: Body mass index
ESS: Epworth Sleepiness Scale
SAMS: Sleep Apnea Multilevel Surgery
COPD: Chronic obstructive pulmonary disease
FEV: Forced expiratory volume
FVC: Forced vital capacity
OAHI: Obstructive apnea hypopnea index
OAI: Obstructive apnea index
CSA: Airway cross-sectional area
SPt: Soft palate
PNS: Posterior nasal spine
Hy: Hyoid bone
RP: Retropalatal
RG: Retroglossal
HIF-1α: Hypoxia inducible factor-1α
REM: Rapid eye movement
