# Supplementary material for: The relationship of tongue fat content and efficacy of uvulopalatopharyngoplasty in Chinese patients with obstructive sleep apnea
Source: BMC Surg. 2023 Aug 27;23:254. doi: 10.1186/s12893-023-02144-x (PMC10463434; doi:10.1186/s12893-023-02144-x)
Supplement: Supplementary file 1 — Additional File 1: Figure s1 and s2 [file 12893_2023_2144_MOESM1_ESM.docx]

Supplementary Materials

Figure S1. The correlations among tongue volume, tongue fat content and clinical factors

95% confidence intervals are plotted on each graph. RG: Retroglossal, BMI: body mass index.

Figure S2. The correlations among tongue volume, tongue fat content, the severity of OSA and ESS

95% confidence intervals are plotted on each graph. AHI: apnea hypopnea index, OAHI: obstructive apnea hypopnea index, OAI: obstructive apnea index, ESS: Epworth Sleepiness Scale.
